# Antimicrobial Nanomaterials Derived from Natural Products—A Review

**DOI:** 10.3390/ma9040255

**Published:** 2016-03-30

**Authors:** Ji Wang, Wilfred Vermerris

**Affiliations:** 1 Department of Microbiology & Cell Science, IFAS, University of Florida, Cancer/Genetics Research Complex 302, 2033 Mowry Road, Gainesville, FL 32610, USA; wangji@ufl.edu; 2 UF Genetics Institute, University of Florida, Gainesville, FL 32610, USA

**Keywords:** antimicrobial properties, biopolymers, chitosan, lignin, nanomaterials

## Abstract

Modern medicine has relied heavily on the availability of effective antibiotics to manage infections and enable invasive surgery. With the emergence of antibiotic-resistant bacteria, novel approaches are necessary to prevent the formation of biofilms on sensitive surfaces such as medical implants. Advances in nanotechnology have resulted in novel materials and the ability to create novel surface topographies. This review article provides an overview of advances in the fabrication of antimicrobial nanomaterials that are derived from biological polymers or that rely on the incorporation of natural compounds with antimicrobial activity in nanofibers made from synthetic materials. The availability of these novel materials will contribute to ensuring that the current level of medical care can be maintained as more bacteria are expected to develop resistance against existing antibiotics.

## 1. Introduction

Modern medicine has undergone revolutionary advances in the past 100 years, which can be attributed in part to the availability of effective antibiotics based on the initial discovery of penicillin by Nobel Prize laureate Dr. Alexander Fleming. Antibiotics have enabled complicated, invasive surgical techniques during which internal organs are exposed to microbes they normally do not encounter. Furthermore, the widespread use of medical implants (e.g., artificial joints) and medical devices (e.g., pacemakers) provide surfaces foreign to the human body to which microbes can attach, which increases the risk of infections and the need for treatment with antibiotics. The widespread use of antibiotics, and especially their misuse in cases where they are not effective (e.g., in treating the common cold or influenza), plus their preemptive use in animal husbandry, has resulted in the appearance of antibiotic-resistant bacteria [1,2]. At the same time, pharmaceutical companies have reduced efforts towards the development of novel antibiotics, in part because of their lower return on investment compared to other kinds of pharmaceutical products. Unless more effort is placed on creating novel antibiotics, the combined effect of these developments is that alternative strategies are needed to ensure future generations can continue to benefit from the advances of modern medicine without serious complications or increasing fatality rates. In addition to developing novel classes of antibacterial compounds to treat infections, mechanisms that reduce the colonizing ability of pathogenic bacteria can contribute to reducing the incidence of infections and associated complications. Developments in material science and engineering, especially in nanotechnology, have resulted in novel materials with properties that lend themselves to use in biomedical applications [3]. With the concomitant rising interest in the use of renewable feedstocks such as woody biomass and crop residues for the production of fuels and chemicals [3,4,5], there are great opportunities for the use of biological materials in medical applications [6]. This review article summarizes different strategies involving the use of nanofibers made from natural polymers and nanofibers containing natural products that have been or are being exploited to reduce microbial infections. The emphasis is on publications from the past five years.

## 2. Antimicrobial Surfaces Limit Bacterial Adhesion or Are Bactericidal

A typical surface is covered with a variety of microbes, some of which may be potentially harmful. In many instances, the microbes can be killed with 70% (*w*/*v*) ethanol or iso-propanol, or a cleansing solution containing a strong detergent, antiseptic compound, or bleach. This is not an option when the surface is sensitive to aforementioned antimicrobial solutions, when it is not practical to clean a given surface on a regular basis, and/or when the surface is difficult to reach. The latter two conditions apply to surfaces inside the human body. For surfaces that cannot be sanitized with routine techniques, it would be most effective to minimize bacterial attachment on the surface, and intense efforts have been devoted to fabricate antimicrobial surfaces. Antimicrobial surfaces can be either anti-biofouling surfaces, which prevent bacterial attachment, or bactericidal surfaces, which allow bacterial attachment but kill the bacteria on contact. Various surface modification approaches have been developed, which can be generally categorized as chemical modification of the surface or physical modification of surface topography. Chemical modification of the surface involves encapsulating or covalently binding antimicrobial agents on the surface. The antimicrobial mode is directly related to the concentration of agents on the surface: while a minimum inhibitory concentration (MIC) is needed to inhibit growth of microorganisms, a minimum biocidal concentration should be met to kill bacteria and fungi. Various antimicrobial agents, such as quaternary ammonium compounds, polybiguanides, halogenated phenols, and polyethyleneimines have been immobilized on surfaces and shown to have adverse effects on bacteria [7,8].

**Figure 1:**
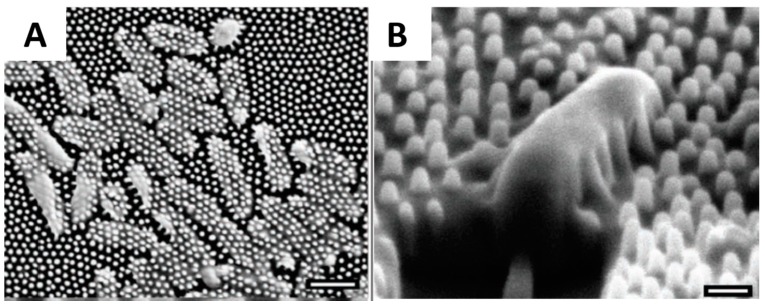
(**A**,**B**) The bactericidal effect of the cicada wing surface on *Pseudomonas aeruginosa*. Cells are clearly penetrated by the nanopolar structures on the wing surface. Scale bar equals 1 µm in (**A**); 200 nm in (**B**). Figures reproduced from [27] with permission from Wiley.

Nanotechnology consists of construction and characterization of nanoscale materials and structures, which may exhibit enhanced physical and chemical properties relative to the bulk materials [9,10]. Anti-microbial nanomaterials have a large surface-area-to-volume ratio, which confers higher active contact surface. Recent advances in nanotechnology have revealed the antimicrobial/antifouling effects of nature-inspired nanoscale topographies, which includes lotus leaves [11,12], cicada wings [13], shark skin [14], feet of gecko [15], wings of dragonflies and butterflies [16,17]. In some studies super-hydrophobicity was hypothesized as a prerequisite to anti-biofouling, as reduced contact area between bacteria and nanostructured surfaces prevents adhesion [18]. Along this line, biomimetic super-hydrophobic surfaces with regular nanoscale topographies have been developed [19,20,21,22]. A closely related and intensively investigated phenomenon is the “lotus effect”, referring to the leaf surfaces of lotus plants, which have binary structures at both microscale and nanoscale, making it possible to trap proportionally large amounts of air and minimize the actual contact area between the water droplets and surfaces [20,23,24,25,26]. Generating nanoscale roughness with materials exhibiting low surface energy is the key factor to constructing these super-hydrophobic surfaces. On the other hand, a mechanism to mechanically kill bacteria was recently discovered on the surface of cicada wings, where arrays of nanopillars penetrate and kill adherent bacteria within several minutes [27] (Figure 1A,B). Inspired by cicada wings, a library of biomimetic nanopillars was prepared on the surface, which was found to have a lower density of adherent bacteria compared to flat films [18,28,29,30]. The geometry of these nanostructures, including the size, spacing and aspect ratio have been demonstrated to be important factors for inhibiting bacterial colonization.

An alternative approach to creating antimicrobial surfaces is the application of nanofiber coatings. With the development of electrospinning, template synthesis and self-assembly approaches, nanofibers have been widely used to incorporate antimicrobial agents, which includes quaternary ammonium compounds [31,32], nanoparticles of noble metals and metal oxides [33,34]. Nanofibers have been shown to be able to control the concentration of released agents and improve the durability and efficiency of the antimicrobial activity [35]. Specific applications will be discussed in later sections of this article.

In recent years, major public concerns have been raised over the effects of synthetic nano-sized antimicrobial compounds on the environment in general, and human health in particular [9,36,37]. Consequently, the use of a number of natural antimicrobial agents is rapidly growing, because they provide antimicrobial surfaces that tend to be non-toxic and environmentally benign. These natural antimicrobial compounds are mostly extracted from plants, and include polysaccharides and their derivatives, peptides, enzymes, essential oils, weak organic acids and polyphenols. These different classes of compounds will be reviewed in terms of their synthesis and efficacy in reducing microbial adhesion.

Whenever novel compounds need to be evaluated for their anti-microbial properties, it is common to monitor their effect on the growth of at least two species of bacteria, with *Escherichia coli* (*E. coli*) and *Staphylococcus aureus* (*S. aureus*) being used most commonly. These bacteria are of particular value because they represent bacteria that are present in large numbers on and in humans. *E. coli* is an important member of the human intestinal microbial community, but certain strains are pathogenic and can create serious health problems when they are present on food and when that food is not adequately prepared [38]. *S. aureus* is present on the skin and in the nasal cavity of 30% of people without any obvious effects, but can also be a serious pathogen, responsible for bacteremia (infection of the blood), endocarditis (infection of the heart), and a variety of other infections [39]. The appearance of methicillin-resistant *S. aureus* (MRSA) is posing serious health risks due to its insensitivity to many antibiotics, with treatment now requiring combinations of multiple synergistic antibiotics [40]. The other reason *E. coli* and *S. aureus* are often used is that they fundamentally differ from each other in the way their outer membranes are organized. *E. coli* is a Gram-negative bacterium, whereas *S. aureus* is Gram-positive. This designation reflects the organization of the bacterial membrane, as it is (positive) or is not (negative) able to retain crystal violet (Gram stain) following an ethanol wash. Gram-positive bacteria have only a single lipid membrane that is surrounded by a thick layer of peptidoglycans, responsible for capturing the crystal violet stain. In contrast, Gram-negative bacteria have an inner and outer membrane separated by a periplasmic space containing a thin layer of peptidoglycans. Since antibiotics need to penetrate the bacterial membrane in order to exert their effects, the interaction with the membrane is an important factor. The results obtained with *E. coli* and *S. aureus* generally provide an indication of how the compound under in investigation will interact with other Gram-positive or Gram-negative bacteria.

## 3. Antimicrobial Nanofibers of Biological Origin

This section of the article summarizes recent results obtained with nanofibers derived from bio-based polymers (chitosan, cellulose, antimicobial proteins) and from synthetic nanofibers containing antimicrobial compounds of biological origin. In all instances, these nanomaterials have been shown to be effective against microbes, and are being utilized to minimize bacterial adherence to surfaces.

### 3.1. Chitosan Nanofibers

Chitosan is a deacetylated derivative of chitin, which is a natural polysaccharide present in the exoskeletons of crustaceans, insects, and certain fungi. It is an abundant waste product of the shellfish industry. Chitin consists of a linear chain of β-1,4-linked *N*-acetyl d-glucosamine residues. The acetyl residues can be removed with enzymes or with alkaline solutions [41,42]. Due to the presence of amino moieties, chitosan becomes polycationic as the environmental pH drops below the polymer’s pK_a_ ~6.5. Their antimicrobial function arises from the electrostatic interaction between the polycationic structure of chitosan and anionic exterior surfaces of microorganisms [43]. Adhesion of bacteria to chitosan results in the disruption of the cell membrane and leakage of intracellular components. Chitosan can also penetrate the bacterial cell membrane and interact with DNA, inhibiting DNA transcription and ultimately protein synthesis [44]. The antimicrobial efficacy of chitosan depends on several factors, including environmental pH, degree of deacetylation and molecular weight.

As an inexpensive natural polysaccharide, chitosan has a wide spectrum of antimicrobial activities against both Gram-positive and Gram-negative bacteria. Besides, chitosan is nontoxic towards mammalian cells and biodegradable. These characteristics make chitosan a desirable polymer for antimicrobial applications and chitosan nanofibers have been the subject of many studies in recent years.

**Figure 2:**
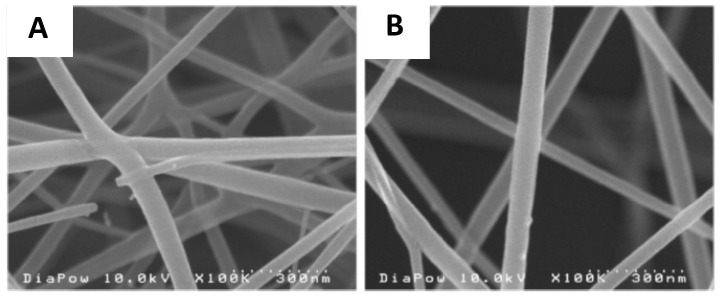
Scanning electron micrographs of electrospun chitosan nanofibers: (**A**) *M_v_* = 158 × 10^4^ g mol^−1^ at 3.25% (*w*/*w*); and (**B**) *M_v_* = 180 × 10^4^ g mol^−1^ at 2.0% (*w*/*w*). Figures reproduced from [50] with permission from ACS.

Electrospinnning is the most commonly used approach to obtain polymer nanofibers. In a typical electrospinning process, an electrical field is used to stretch a viscoelastic polymer solution jet, which solidifies into nanofibers and can be collected as nonwoven mats [45]. In terms of the flexibility of the process, electrospinning is able to fabricate nanofibers from a wide variety of polymers. However, electrospinning of chitosan is challenging. Due to the polycationic nature and the hydrogen bonding between molecules, chitosan has limited solubility in common organic solvents [46,47,48]. While acidic aqueous solutions can be used to dissolve chitosan, chitosan solutions are often difficult to electrospin due to their high viscosity and surface tension. Pure chitosan nanofibers have only been successfully electrospun from 7% to 8% (*w*/*w*) solutions with low molecular weight chitosan. For example, Geng *et al.* [49] electrospun nanofibers from a 7% (*w*/*w*) solution of 106-kDa chitosan dissolved in 90% (*v*/*v*) acetic acid. Another successful attempt at electrospinning of pure chitosan was reported by Ohkawa *et al.* [50], who obtained smooth nanofibers by dissolving chitosan in trifluoroacetic acid. Homogenous nanofiber networks were obtained under optimized solution concentration and chitosan molecular weights (Figure 2). In antimicrobial tests, a significant reduction of *S. aureus* colonies (>99.9%) was observed after 24 h of incubation in the presence of chitosan nanofiber mats [51].

To increase the solubility of chitosan, chitosan derivatives have been investigated. Alkyl groups were introduced onto the amine groups of chitosan. Subsequent reaction with methyl iodide produced water-soluble quaternary salts of chitosan [52]. Compared with pure chitosan, these quaternary chitosan derivatives demonstrated higher antimicrobial activity. Jia *et al.* [53] reported that the MIC of *N*-*N*-propyl-*N*,*N*-dimethyl chitosan against *E*. *coli* is 20 times lower than that of pure chitosan. Electrospun nanofibers have been fabricated using quaternized chitosan derivatives blended with biodegradable polymers, such as poly(lactic acid) (PLA), poly(vinyl pyrrolidone) (PVP) and poly(vinyl alcohol) (PVA). Ignatova *et al.* [54] prepared PLA/quaternized chitosan nanofibers at a weight ratio of quaternized chitosan: PLA equal to 30:70. The hybrid nanofibers were further crosslinked with glutaraldehyde for stabilization in water and the crosslinked nanofiber mats demonstrated the capability to kill all the *S. aureus* and *E. coli* bacteria within 60 min of contact. The same group also reported preparation of PVA/quaternized chitosan nanofibers, which were crosslinked with triethylene glycol diacrylate (TEGDA) under UV light. The resultant nanofiber mats containing 2.9 g quaternized chitosan showed a 98% reduction in the number of *E. coli* bacteria after 120 min contact time [55]. Similarly, Alipour *et al.* [56] prepared crosslinked PVA/quaternized chitosan nanofibers at different PVA: quaternized chitosan ratios, and reported that, when the crosslinked nanofiber mats were in contact with *S. aureus*, the diameter of inhibition zones increased with increasing amount of quaternized chitosan in the hybrid nanofibers. In addition, quaternized chitosan nanofibers have been prepared from other soluble derivatives, such as hexanoyl chitosan [57], carboxymethyl chitosan [58] and poly(ethyleneglycol)-grafted chitosan [59]. By manipulating the surface tension, conductivity and viscosity of the spinning solution, smooth and uniform fibers were obtained over a wide range of diameters (40 nm–4 µm) [60].

**Figure 3:**
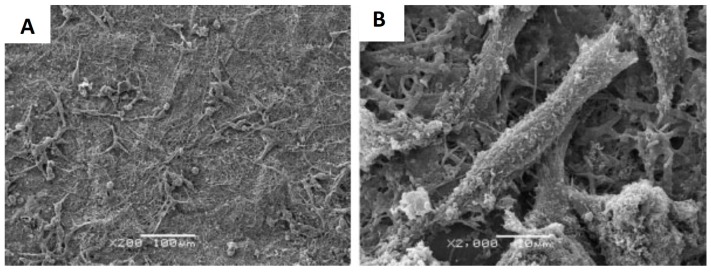
Scanning electron micrographs of mouse fibroblasts seeded on fibrous membranes of hydroxyapatite containing chitosan/polyvinyl alcohol after 48 h culture. (**A**) 200 × magnification; (**B**) 2000 × magnification. Figures reproduced from [65] with permission from Wiley.

In parallel with the development of chitosan derivatives, it was shown that producing nanofibers from chitosan solutions by electrospinning is feasible, provided a flexible second polymer is present. Commonly used fiber-forming polymers include PVA and polyethylene oxide (PEO), which are non-toxic, biocompatible and biodegradable. By replacing some of the chitosan with PEO or PVA in solution, the overall viscosity and surface tension could be reduced, which facilitated the electrospinning process [61]. Desai *et al.* [62] fabricated PEO/chitosan nanofibers as filter media and the chitosan blend fibers demonstrated a 100–1000 fold reduction of *E. coli* after 6 h of contact. Iraj *et al.* [63] prepared chitosan-PEO nanofibers loaded with 0.25% (*w*/*w*) silver nanoparticle, which demonstrated 100% inhibition against both *E. coli* and *S. aureus*. Chitosan/PVA and chitosan/PEO nanofiber scaffolds are of particular interest for tissue engineering applications as they were shown to promote cell attachment and growth while inhibit bacteria growth. The potential applications of these biocomposite nanofiber scaffolds for tissue repair/regeneration have been demonstrated with human chondrocytes, osteoblasts [64], and mouse fibroblasts [65] (Figure 3). Recently, chitosan nanofibers were created as wound dressings for burn healing and the *in vivo* results showed that the chitosan nanofiber dressing provide effective protection from infections while stimulating the process of skin tissue regeneration [66]. Similarly, composite nanofiber scaffolds made from chitosan and silk fibroin demonstrated antimicrobial activities against *E. coli* and *S. aureus* while promoting fibroblast attachment and proliferation [67].

### 3.2. Cellulose Nanofibers

Cellulose is the most abundant natural polymer on Earth, with a long history of use in fiber fabrication [68]. Nowadays, electrospun nanofibers made from cellulose and its derivatives, such as cellulose acetate and cellulose hydroxyl propyl, are finding wide use in antimicrobial applications. Cellulose and its derivatives do not intrinsically have antimicrobial properties. To achieve antimicrobial properties, antimicrobial agents, such as sorbic acid [69], benzalkonium chloride [70], copper nanoparticles [71] and silver chloride nanoparticles [72] have been successfully incorporated into cellulose nanofibers. In addition, antimicrobial cellulose membranes can be obtained by chemically grafting functional groups onto the surface of cellulose nanofiber network. Roy *et al.* [73] immobilized quaternary ammonium groups onto cellulose nanofibers through reversible addition-fragmentation chain transfer (RAFT). Recently, researchers have successfully prepared cellulose nanofibers functionalized with amino groups [74,75] and aminosilane groups [76], which effectively inhibited growth of *S. aureus* and *E. coli*.

### 3.3. Nanofibers Containing Antimicrobial Peptides (AMPs)

Antimicrobial peptides (AMPs) are functionally defined, as their name implies. AMPs have been isolated from a wide variety of sources, including plants, animals, bacteria and viruses [37]. Despite the diversity of their sources, AMPs share some common features in their structures and functionalities. They are generally between 12 and 50 amino acid in length, with two or more cationic amino acid residues and a substantial proportion of hydrophobic moieties. Thus far, more than 2000 AMPs have been reported in the antimicrobial peptide database [77]. AMPs are key components of the innate immune systems of animals and play important roles in early defense against invading microorganisms. Compared with conventional antibiotics, AMPs demonstrate unique features such as low propensity for inducing the formation of resistant pathogens, the ability to discriminate host and invading cells, and high activity against a broad spectrum of microorganisms. The positive charge facilitates the initial association of AMPs with negatively charged cell membranes, while their hydrophobicity allows subsequent insertion of AMPs into the hydrophobic cores of the cell membrane, resulting in membrane rupture and cell lysis. Since AMPs target bacterial membranes, which are difficult to redesign from non-lipid molecules, it will likely be difficult for microorganisms to develop resistance to AMPs [78]. Thus, AMPs have become promising candidates to replace traditional antimicrobial agents.

In order for AMPs to function properly, they must be delivered in a way that maintains their efficiency. Recently, electrospun nanofibers were used as carriers for AMPs, which provide large surface areas and extended contact time, efficiently controlling release profiles, as well as protection from proteolytic enzymes. By electrospinning the AMP nisin into PEO/poly (d,l-lactide) (PDLLA) nanofibers, Heunis *et al.* [79] fabricated an antimicrobial wound dressing. Active nisin diffusion lasted for four days *in vitro*. The cell number of *S. aureus* at the wound site decreased by more than 99.9%. The same group also reported incorporation of AMP ST4SA (a bacteriocin produced by *Enterococcus mundtii*) into nanofibers [80]. The AMPs retained 88% of their original antimicrobial activity after 18 h of incubation and application of 25 mg nanofibers inhibited the growth of *Enterococcus faecium* HKLHS for up to 10 h. For the preparation of antimicrobial surfaces, PVA and PEO nanofibers are most commonly used for AMP loading. Viana *et al.* [81] incorporated *Centritchis muricatus* membrane permeation peptide 1 (Cm-p1) into PVA nanofibers with diameters of 500–600 nm. The Cm-p1 release behavior from the nanofibers continued for three days, leading to effective *Candida albicans* (*C. albicans*) control in the first 24 h. In addition, the nanofibers did not affect mammalian cell viability, demonstrating the biocompatibility of the nanofibers as potential wound dressings. Similarly, Torres *et al.* [82] prepared PVA-based subtilosin nanofibers of 270-nm diameter via electrospinning. The loaded PVA nanofiber (2.4 mg subtilosin/g fiber) inhibited wild-type Herpes Simplex Virus type 1. At the same time, Gatti *et al.* [83] prepared PEO nanofibers loaded with the bacteriolytic antibody LL-37. The AMP-loaded PEO nanofibers could eliminate bacteria in their immediate vicinity and could eliminate colonies as culture medium passed through the fiber mesh. Recently, Yüksel and Karakeçili [84] reported covalent immobilization of Magainin II, which is a 23-residue AMP, on poly(lactic-co-glycolic acid) (PLGA)-electrospun nanofibers. The bound Magainin II demonstrated antimicrobial activity against both *E. coli* and *S. aureus*. After 4 h of incubation, Magainin II reduced the number of adhered bacteria by more than 50%.

It should be noted that the antimicrobial activity of AMPs can be significantly affected when incorporated into nanofibers. Sebe *et al.* [85] reported that the antimicrobial activity of colistin sulfate was improved by 5–6 fold against *Acinetobactor baumannii* and *S. aureus* after incorporation into PVA nanofibers. On the other hand, Eriksen *et al.* [86] incorporated several different synthetic AMPs into poly ɛ-caprolactone (PCL) nanofibers and found that only tetracycline hydrochloride retained its activity. A parallel study by Heunis *et al.* [87] also demonstrated that antimicrobial activity of plantaricin 423 decreased from 51,200 to 25,600 arbitrary units mL^−1^ after electrospinning into PEO nanofibers.

**Figure 4:**
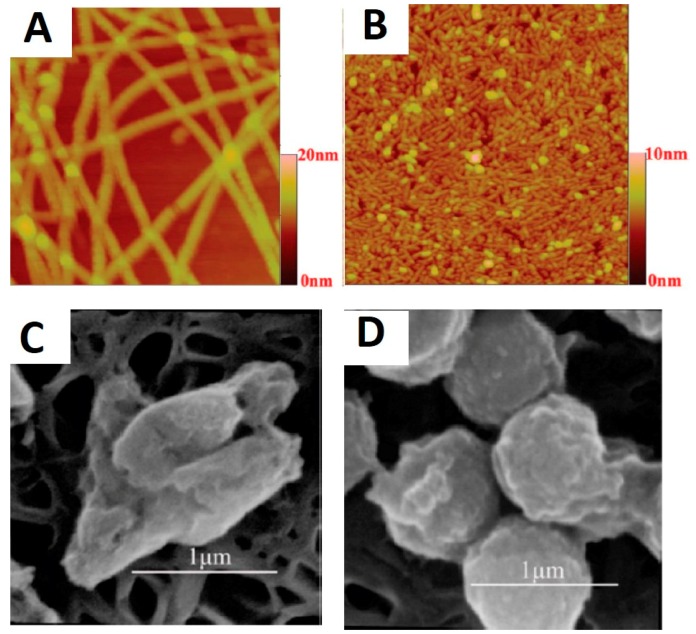
Nanostructures formed by peptide self-assembly as revealed by topographical AFM micrographs formed by: (**A**) A_6_K; and (**B**) A_9_K. Scanning electron micrographs of: (**C**) *E. coli*; and (**D**) *S. aureus* treated by A_9_K. Figures reproduced from [88] with permission from ACS.

Some AMPs display the propensity for nano-aggregation due to the existence of hydrophobic moieties, thus allowing fabrication of AMPs nanofibers directly through self-assembly. Using this approach, Chen *et al.* [88] developed self-assembled nanofibers and nanorods from short synthetic amphiphilic peptides A_6_K and A_9_K respectively, where A denotes hydrophobic alanine and K denotes charged lysine (Figure 4). By increasing the hydrophobic moieties, the peptide assemblies transit from long nanofibers formed by A_6_K to short nanorods by A_9_K. A_9_K exerted its bactericidal capacity through perturbing bacterial membranes, which was followed by bacterial surface collapse and cell lysis. At a concentration of 0.1 mg/mL, the A_9_K nanorods killed 80% of the *E. coli* cells and 70% of the *S. aureus* cells after 1 h incubation.

### 3.4. Antimicrobial Synthetic Nanofibers Containing Plant-Derived Compounds

Plant-derived antimicrobial agents such as propolis and essential oils have been intensively investigated and recent studies have demonstrated that these agents can also be incorporated into nanofibers.

Propolis is a resinous substance produced by honeybees and can be considered a complex mixture containing flavonoid aglycones, phenolic acids and aldehydes, steroids, amino acids as well as natural pigments such as chlorophyll and carotenoids. The antibacterial activity of propolis has been intensively reported and part of the antimicrobial activity is attributed to the phenolic compounds of the flavonoid fractions [89,90]. Kim *et al.* [91] successfully electrospun propolis/polyurethane (PU) nanofibers and observed that nanofiber membranes containing 30% (*w*/*w*) propolis showed the highest inhibitory effects against *E. coli*. They showed that constituents such as flavonoids and cinnamic acid derivatives in propolis were responsible for the antibacterial effects. More recently, Sutjarittangtham *et al.* [92] created PCL nanofibers with a Brazilian source of propolis that was shown to inhibit human pathogenic bacteria in a dosage-dependent manner, with higher concentrations of propolis extract in the nanofibers resulting in a greater degree of inhibition. Nanofiber mats containing 2% (*w*/*w*) of this propolis source exhibited inhibitory effects on *S. aureus*, *Staphylococcus epidermidis*, *Proteus mirabilis* and *E. coli*, whereas at a concentration of 4% (*w*/*w*), *Bacillus cereus* was also inhibited. A challenge with the use of propolis is that its exact composition varies according to local climate, season and flora, making it difficult to maintain uniformity or consistency.

Essential oils are concentrated hydrophobic liquids containing volatile compounds extracted from plants, often with a specific aroma. Hydrophobic terpenoid and phenolic compounds present in essential oils can permeate cell membranes and lead to depletion of protons, disruption of adenosine triphosphate (ATP) synthesis, and in some cases cell lysis. For centuries, essential oils have been used to fight against bacterial infections and because of their mode of action, it is difficult for the bacteria to develop resistance to these volatile antimicrobial agents [93]. Recently, Espina *et al.* [94] demonstrated that individual constituents from essential oils, such as carvacrol, citral and limonene can effectively inhibit the biofilm mass production in community-associated methicillin-resistant *S. aureus* (CA-MRSA). Incorporation of essential oil into nanofiber mats involves electrospinning an oil-in-water emulsion, and stabilizers are usually needed to keep the essential oil drops from coalescing. In 2009, Kriegel *et al.* [95] were the first to successfully prepare essential-oil-containing nanofibers by solubilizing eugenol in micelles made from the surfactant Surfynol 465 and electrospinning the eugenol-containing micro-emulsions mixed with PVA solution. Recently, Rieger and Schiffman [96] successfully incorporated cinnamaldehyde, another volatile essential oil, into PEO nanofibers by using chitosan as stabilizer. The intrinsic antibacterial activity of chitosan along with the quick release of cinnamaldehyde resulted in 80% of *E. coli* being killed within 30 min of bacteria-nanofiber contact.

## 4. Novel Bio-Based Materials with Antimicrobial Properties

This next section of the article describes developments involving biological materials with antimicrobial properties that show promise but that do require additional research before they are ready to be deployed. The approaches include surface modification with known antimicrobial compounds, addition of antimicrobial compounds on nanofibers of biological origin, and fabrication of antimicrobial surfaces with topographies mimicking antimicrobial surfaces found in nature.

### 4.1. Novel Uses of Biological Materials with Known Antimicrobial Properties

Lignin is a phenolic plant cell wall polymer that provides structural support, a mechanical barrier against plant pathogens, and facilitates water transport through xylem vessels because of its hydrophobic nature [97]. Lignin is synthesized via oxidative coupling of monolignols, which are hydroxycinnamyl alcohols and related compounds generated via the shikimic acid and general phenypropanoid pathways [98]. The three principal monolignols are *p*-coumaryl alcohol, coniferyl alcohol and sinapyl alcohol, which, following polymerization, give rise to *p*-hydroxyphenyl, guaiacyl, and syringyl residues, respectively,. The exact chemical structure of native lignin varies among different plant species and between tissues within a given species. Industrial, chemically modified lignin is generated in large quantities by the pulp and paper industry, and by biorefineries that generate renewable fuels and chemicals from the polysaccharides in plant biomass [99,100]. Native lignin with guaiacyl and syringyl structures has been known for its inhibitory effects on microbes since the 1970s [101]. Nada *et al.* [102] showed that lignins precipitated from pulping liquor were also effective against Gram-positive bacteria, such as *Bacillus subtilis* (*B. subtilis*) and *B. mycoides*, but have no antimicrobial effects against Gram-negative bacteria such as *E. coli*. Similarly, Dong *et al.* [103] extracted lignin from corn stover residue using an alkaline solution and showed that the extracts were effective against Gram-positive bacteria (*S. aureus, Listeria monocytogenes*, and *Candida lipolytica*), but not Gram-negative bacteria (*E. coli* and *Salmonella enteritidis*). This implies the antimicrobial effects of lignin are based on its interaction with the thick peptidoglycan layer common to Gram-positive bacteria. Individual phenolic compounds, in contrast, appear to exert their antimicrobial activity using a mechanism that is dependent on uptake through the membrane, and are effective against both Gram-positive and Gram-negative bacteria. Ferulic acid appears to be especially effective compared to related hydroxycinnamic acids and aldehydes (sinapic acid, coniferaldehyde, sinapaldehyde), exhibiting a higher inhibitory efficiency attributed to its increased membrane permeability [104], and a reported MIC of less than 8.0 mM against a wide spectrum of bacteria and yeast, including *B. subtilis*, *E. coli*, *Pseudomonas syringae*, *Saccharomyces cerevisiae*, *Schizosaccharomyces pombe*, and *Sporidiobolus pararoseus*.

Given the abundance of lignin as a low-cost residue from several industrial processes, much effort has been focused on developing value-added products, including in various polymers [98,105,106]. Lignin-containing antimicrobial fibers were first reported by Johnston and Nilsson [107], who prepared nanosilver/lignin/cellulose composite fibers, which inhibited *S. aureus* growth at very low levels of silver (0.008% (*w*/*w*)). Recently, lignin-based nanotubes and nanowires were successfully synthesized by Caicedo *et al.* [108] in a sacrificial alumina membrane template. By covalently adding layers of dehydrogenation polymer onto the base lignin layer within the pores of alumina membrane template, nanotubes with a wall thickness of about 15 nm or nanowires with a diameter around 200 nm were synthesized. Lignin nanotubes (LNTs) are flexible and can be bio-functionalized easily. *In vitro* experiments revealed that these LNTs can enter HeLa cells without auxiliary agents and that depending on the source of the lignin, LNTs can also penetrate the cell nucleus [109]. It is anticipated that LNTs can serve to inhibit microbes, either as free structures following decoration of the outer surface with antimicrobial compounds, or, when adhered to a solid surface, as an antimicrobial coating.

Hundreds of herbal medicines have been used for centuries as treatments against bacterial infections in Asia, and they continue to be used nowadays, although the mode of administering them is becoming more diverse as some of these herbal medicines get incorporated into nanofibers. For example, Nirmala *et al.* [110] incorporated baicalein, which is a flavonoid isolated from the roots of two species of skullcaps (*Scutellaria baicalensis* and *Scutellaria lateriflora*), herbs of the mint family, into PVA nanofibers. The composite nanofibers exhibit inhibitory effects against both *E. coli* and *S. aureus*, and the inhibitory effects became more pronounced as baicalein content increased. In another study, shikonin, which is a major component of the dried root of the Chinese herb *Lithospermum erythrorhizon* (common English name: purple gromwell) was loaded into PCL/poly(trimethylene carbonate) (PTMC) nanofibers [111]. The drug release behavior could be adjusted by varying the PCL: PTMC ratio. After 24 h of contact, 15-mm diameter mats of 5% (*w*/*v*) shikonin-loaded nanofibers displayed a 21.3- and 16.9-mm diameter inhibitory zone for *S. aureus* and *E. coli*, respectively. Efforts were also devoted to produce nanoscale powdered herbal medicines. The size reduction can enhance the antimicrobial activity and allows the possibility of encapsulation into nanofibers. Bhawana *et al.* [112] prepared nanoparticles (diameter 2–40 nm) of curcumin, which has been known for centuries as a household remedy against bacterial infections. Compared with traditional curcumin, nanocurcumin displayed a lower minimal inhibitory concentration (MIC) and larger zone of inhibition, which was attributed to the better dispersion of the compound in aqueous solution. Similarly, Zhang *et al.* [113] prepared aloe vera-conjugated silver nanoparticles with an average diameter of 25 nm. Compared with unmodified silver nanoparticles and aloe vera gel, the aloe vera conjugated to silver nanoparticles demonstrated higher antimicrobial activity, with a MIC of 200 µmol/L. Recently, novel antimicrobial plant extracts have been isolated from a wide variety of plants, such as date palm (*Phoenix dactylifera*) [114], the parasitic plant *Cytinus hypocistis* [115], and *Eurycoma longifolia* [116], a plant native to Southeast Asia, known locally as tongkat ali and used as a nutritional supplement. Extracts from these plants provide additional compounds with potential medicinal properties that can be loaded onto nanofibers.

While some bacteria form colonies, others form biofilms in which cells are physically tightly associated inside an extracellular matrix [117]. Infections arising from biofilm-forming bacteria tend to be more difficult to treat, in part because the inner layer of bacteria is protected from the effects of the antimicrobial compounds that will first attack the outer layer. Biofilm-dispersing enzymes, such as Dispersin B, DNase I, alginate lyase, Proteinase K, trypsin and Serratiopeptidase degrade various biopolymers involved in cell attachment, thus disrupt biofilm formation [37]. Trepat *et al.* [118] demonstrated that bacteria became more susceptible to antimicrobial agents after biofilm dispersion. To immobilize biofilm-dispersing enzymes onto surfaces, various approaches have been developed, including covalent attachment, physical entrapment, ionic interactions and non-covalent adsorption [119]. Several studies have highlighted the feasibility of immobilized enzymes as an efficient approach to prevent bacterial colonization. Shah *et al.* [120] demonstrated that the the antibacterial activity of lysostaphin, an endopeptidase from *Staphylococcus simulans*, when adsorbed to the surface of catheters, could be maintained for at least four days. Recently, Pavlukhina *et al.* [121] reported a reduction in *S. epidermidis* biofilm of 98% upon contact with immobilized enzyme Dispersin B. Simiarly, Yuan *et al.* [122] developed an antifouling surface by immoblizing lysozyme; the resultant surfaces exhibited high antimicrobial efficacy against both *S. aureus* and *E. coli*. There are no reports yet about the incorporation of biofilm-dispersing enzymes into nanofibers. One reason could be that it is difficult to maintain the activity of enzymes during the electrospinning process. Given the success with incorporating other bio-active compounds (e.g., AMPs), it is conceivable that nanofibers loaded with active biofilm-dispersing enzymes will be developed in the near future and serve as efficient antibacterial compounds.

### 4.2. Biopolymers as Carriers of Antimicrobial Agents

Zein is the prolamine (plant storage protein with a high content of α-amino acids) in maize (*Zea mays*) and an abundant biodegradable and renewable polymer. Zein possesses excellent solubility in alcohols and good elasticity and film-forming capability. Recently, electrospun zein nanofibers have been utilized in antimicrobial applications. De Oliveira Mori *et al.* [123] successfully incorporated tannin from bark extracts of the leguminous shrub *Stryphnodendron adstringens* into zein nanofibers by dissolving both zein and tannin in a water/alcohol mixture. Similarly, Unnithan *et al.* [124] prepared polyurethane-cellulose acetate-zein composite nanofiber mats for wound dressing. After incorporation of streptomycin sulfate, the composite nanofiber mats demonstrate excellent bactericidal activity against a wide range of bacteria, including *E. coli*, *S. aureus*, *B. subtilis*, *Salmonella typhimurium*, and *Vibrio vulnificus*.

Cyclodextrins are a family of cyclic oligosaccharides with a toroid-shaped molecular structure, which can form host-guest complexes with a variety of molecules through non-covalent bonds. Cyclodextrins are capable of self-assembly through intermolecular interactions, such as hydrogen bonding, Van der Waals forces and charge transfer. Celebioglu and Uyar [125] demonstrated successful electrospinning of cyclodextrins and reported that electrospinning hydroxypropyl-β-cyclodextrin/triclosan solution with 1:1 molar ratio yields nanofibers. Recently, cyclodextrin-associated antimicrobial agents were incorporated into nanofibers. Aytac *et al.* [126] incorporated an inclusion complex of allyl isothiocyanate (AITC) with β-cyclodextrin into PVA nanofibers. As a result, sustained release of AITC was achieved and the nanofiber mats demonstrated inhibitory activity against *E. coli* and *S. aureus*. Celebioglu *et al.* [127] prepared silver nanoparticles containing PVA nanofibers, and hydroxypropyl-β-cyclodextrin was utilized to reduce the size of Ag nanoparticles. The Ag particle size was reduced from 8 to 2 nm, which improved the antimicrobial activity of the nanofiber mats.

Natural biodegradable polymers such as soy protein, sericin and alginates can also be utilized in antimicrobial nanofiber fabrications. Soy protein is renewable, economical and biocompatible. In 2009, Vega-Lugo and Lim [128] incorporated AITC into soy protein/PEO electrospun fibers and reported that release of the antimicrobial AITC increased substantially as relative humidity increased. Similarly, Xu *et al.* [129] prepared soy protein/PEO hybrid nanofibers by dissolving both components in 1,1,1,3,3,3-hexafluoro-2-propanol (HFIP). The diameter of the resulting fibers was in the range of 200–300 nm. A different procedure to produce soy protein nanofibers was employed by Zhang *et al.* [130]. They used a solution blowing approach and incorporated silver nanoparticles. The resulting nanofibers, demonstrated significant antibacterial activity against *E. coli* colonies. Silk sericin protein is 20%–30% (*w*/*v*) of the silkworm cocoon filament and needs to be removed to get good luster and hand feel of silk fabrics. The removed silk sericin is widely used in medical applications and tissue engineering, because it demonstrates excellent biocompatibility and oxidation resistance. Zhang *et al.* [131] and Khan *et al.* [132] obtained sericin nanofibers by electrospinning silk sericin solution in trifluoroacetic acid (TFA). Sericin was also used to in combination with chitosan to form composite nanofibers that could be used a wound dressing [133]. Concurrently, Hadipour *et al.* [134] demonstrated successful electrospinning of chitosan/sericin/PVA nanofibers containing nanosilver particles. Alginate is a polymer derived from seaweed and has been extensively studied owing to its biocompatibility. In 2006, Bhattarai and Li [135] successfully developed alginate/PEO nanofibrous scaffolds with an average fiber diameter of around 70 nm. Recently, Shalumon *et al.* [136] and Liakos *et al.* [137] prepared antimicrobial sodium alginate nanofiber mats containing nano-ZnO and essential oil, respectively. In addition to the above mentioned biopolymers, nanofibers made from wheat protein [138], starch [139] and milk protein [140] and have been developed in the context of tissue engineering and biomedical applications, providing more candidate materials that can be loaded with antimicrobial agents.

### 4.3. Mimicking Naturally Occurring Antimicrobial Surfaces with Novel Fabrication Techniques

As mentioned earlier, one of the approaches to obtaining antibacterial properties is to carefully design the surface topography. The ability to create specific surface topographies has been facilitated by the development of novel nanofiber fabrication techniques. Cicada wing surfaces were found to be to deadly to microbial cells, and the bactericidal effects were attributed to the physical surface features, whereby nanopillars on the surface penetrated the cells [27]. These observations led to the fabrication of synthetic surfaces that mimicked these naturally occurring antimicrobial surfaces. Inspired by the cicada wing surfaces, Diu *et al.* [141] engineered surfaces with TiO_2_ nanowire arrays through an alkaline hydrothermal process. The engineered surfaces were reported to be selectively bactericidal against motile bacteria, while favoring mammalian cell growth. Similarly, Tiraferri *et al.* [142] covalently bound single-walled carbon nanotubes to polyamide membranes, which resulted in 60% inactivation of bacteria within 1 h of contact. Using a recently developed spinneret-based tunable engineered technique, Kargar *et al.* [143] achieved polystyrene nanofiber patterns with precisely controlled fiber diameter and fiber spacing, which provided antifouling effects against *Pseudomonas aeruginosa*.

Current progress towards sub-100-nm-diameter nanofibers [144,145,146,147,148] with controlled morphologies, such as porous fiber [149,150,151], wrinkled fiber [152,153] and hollow fiber [154,155,156,157,158] provides nanofibers with enhanced specific surface area, which may further improve their biocidal efficiency.

## 5. Future Prospects

While novel antimicrobial agents, biopolymers and novel nanofiber fabrication techniques provide new opportunities for the development of antimicrobial nanofibers, some important challenges need to be kept in mind while pursuing these. First, the inherent genetic flexibility of microbial populations provides mechanisms for bacteria to develop antibiotic resistance through either mutation of target sites or acquisition of novel biochemical functions [159]. Although there is intensive research on bacterial resistance towards traditional antimicrobial compounds such as silver and quaternary ammonium compounds, research is also needed to investigate the potential of microbes developing resistance towards the novel antimicrobial agents described in this article, such as chitosan and AMPs, especially after their immobilization onto nanofibers. Knowing the longer-term efficacy of these novel materials is important in order to prevent widespread infections in patients relying on these materials. When considering antimicrobial nanofibers, it is important to consider that evidence supporting the high biocidal efficiency of a novel antimicrobial agent cannot automatically be extrapolated to predict their efficacy after their immobilization onto nanofibers. This is illustrated by the reports on reduced biocidal efficiency of AMPs after incorporation into nanofibers [80,86]. Hence, for applications involving medical implants, an extensive *in vivo* evaluation of the durability and stability of these nanofibers-based antimicrobial surfaces is needed. In particular, the accumulation of dead bacteria on the antimicrobial surfaces may promote more bacterial accumulation thus reducing the antimicrobial activities over time [37]. Development of surfaces of both antimicrobial and antifouling properties may be a strategy to address this problem.

In summary, significant progress has been made in the development of bio-based antimicrobial nanofibers, and novel biocidal agents and biopolymers present a variety of candidates for the next generation of non-toxic and biodegradable antimicrobial nanofibers. With further research to evaluate their longer-term *in vivo* performance, such as antibiotic resistance, stability and durability, these materials offer the prospect of contributing to a continued high level of medical care in decades to come.
